# Simpson Rubber vs. Goodyear Rubber

**Published:** 1868-04

**Authors:** 


*Simpson Rubber vs. Goodyear Rubber.*—The following result of a recent suit in the U. S. Circuit Court of the Northern District of New York may prove of interest to many of your readers.

"In the case of Henry B. Goodyear Dental Vulcanite Co. *vs.* Dr. J. Brockway, of Albany,—which was a suit for using hard Rubber for Dental purposes, before the U. S. Circuit Court for the Northern District of New York,—an application for a preliminary injunction to restrain the Doctor from further use of the article pending the suit was set down for a hearing on Tuesday, Jan. 27, at Albany. The motion was resisted by Defendant on the ground that he was using the Simpson Dental Rubber, and not the Goodyear Rubber, or any infringement of it. Chas. F. Blake, Esq., of New York, appeared for the owners of the Goodyear Patents, and H. T. Blake, Esq., of Bridgeport, for the owners of the Simpson Patent. Affidavits had been prepared on behalf of the Defendant, disclosing the fact that the Simpson Rubber is entirely different, both as a material and in its mode of manufacture, from the Goodyear article, upon the exhibition of which the counsel for the Complainants notified the Court that he should decline to press the motion at present, as he had intended and requested and obtained an indefinite postponement of the whole matter."

